# Somatic PDGFRB activating variants promote smooth muscle cell phenotype modulation in intracranial fusiform aneurysm

**DOI:** 10.1186/s12929-024-01040-7

**Published:** 2024-05-13

**Authors:** Li Hao, Xiaolong Ya, Jiaye Wu, Chuming Tao, Ruochen Ma, Zhiyao Zheng, Siqi Mou, Yiming Ling, Yingxi Yang, Jiguang Wang, Yan Zhang, Qing Lin, Jizong Zhao

**Affiliations:** 1 Department of Neurosurgery, Beijing Tiantan Hospital, Capital Medical University, Beijing, 100070 China; 2 China National Clinical Research Center for Neurological Diseases, Beijing, 100070 China; 3 Joint Laboratory of School of Pharmacy, Capital Medical University and National Clinical Research Center for Nervous System Diseases, Beijing, China; 4 Division of Cell, Developmental and Integrative Biology, School of Medicine, South China University of Technology, Guangzhou, 510006 China; 5 HKUST Shenzhen-Hong Kong Collaborative Innovation Research Institute, Futian, Shenzhen, China; 6 Peking Union Medical College Hospital, Chinese Academy of Medical Sciences and Peking Union Medical College, Beijing, 100730 China; 7 University of Chinese Academy of Sciences, Beijing, 100049 China; 8 Department of Chemical and Biological Engineering, The Hong Kong University of Science and Technology, Hong Kong, China; 9 Department of Chemical and Biological Engineering, Division of Life Science, State Key Laboratory of Molecular Neuroscience, The Hong Kong University of Science and Technology, Hong Kong SAR, China; 10 Hong Kong Center for Neurodegenerative Diseases, InnoHK, HKSAR, China

**Keywords:** PDGFRB variants, Phenotype modulation, Intracranial fusiform aneurysm, Smooth muscle cell

## Abstract

**Background:**

The fusiform aneurysm is a nonsaccular dilatation affecting the entire vessel wall over a short distance. Although PDGFRB somatic variants have been identified in fusiform intracranial aneurysms, the molecular and cellular mechanisms driving fusiform intracranial aneurysms due to PDGFRB somatic variants remain poorly understood.

**Methods:**

In this study, single-cell sequencing and immunofluorescence were employed to investigate the phenotypic changes in smooth muscle cells within fusiform intracranial aneurysms. Whole-exome sequencing revealed the presence of PDGFRB gene mutations in fusiform intracranial aneurysms. Subsequent immunoprecipitation experiments further explored the functional alterations of these mutated PDGFRB proteins. For the common c.1684 mutation site of PDGFRβ, we established mutant smooth muscle cell lines and zebrafish models. These models allowed us to simulate the effects of PDGFRB mutations. We explored the major downstream cellular pathways affected by PDGFRB^Y562D^ mutations and evaluated the potential therapeutic effects of Ruxolitinib.

**Results:**

Single-cell sequencing of two fusiform intracranial aneurysms sample revealed downregulated smooth muscle cell markers and overexpression of inflammation-related markers in vascular smooth muscle cells, which was validated by immunofluorescence staining, indicating smooth muscle cell phenotype modulation is involved in fusiform aneurysm. Whole-exome sequencing was performed on seven intracranial aneurysms (six fusiform and one saccular) and PDGFRB somatic mutations were detected in four fusiform aneurysms. Laser microdissection and Sanger sequencing results indicated that the PDGFRB mutations were present in smooth muscle layer. For the c.1684 (chr5: 149505131) site mutation reported many times, further cell experiments showed that PDGFRB^Y562D^ mutations promoted inflammatory-related vascular smooth muscle cell phenotype and JAK-STAT pathway played a crucial role in the process. Notably, transfection of PDGFRB^Y562D^ in zebrafish embryos resulted in cerebral vascular anomalies. Ruxolitinib, the JAK inhibitor, could reversed the smooth muscle cells phenotype modulation in vitro and inhibit the vascular anomalies in zebrafish induced by PDGFRB mutation.

**Conclusion:**

Our findings suggested that PDGFRB somatic variants played a role in regulating smooth muscle cells phenotype modulation in fusiform aneurysms and offered a potential therapeutic option for fusiform aneurysms.

**Supplementary Information:**

The online version contains supplementary material available at 10.1186/s12929-024-01040-7.

## Introduction

Intracranial aneurysms can be categorized into two primary types based on their morphological characteristics: saccular and nonsaccular [1]. Fusiform aneurysms, a subtype of nonsaccular aneurysms, are characterized by circumferential dilatation of the vessel wall over a short segment. Representing approximately 3%-13% of all intracranial aneurysms, fusiform aneurysms predominantly occur within the vertebrobasilar system [2–5]. Patients with intracranial fusiform aneurysms frequently exhibit symptoms and signs indicative of occlusion, arterial rupture, or mass effect [6–8]. Surgical or endovascular treatment of fusiform aneurysms presents a significant challenge due to the associated risks and complexities.

In comparison to saccular aneurysms, fusiform aneurysms exhibit distinct pathologies, hemodynamics, anatomical distributions, natural histories, and treatment modalities, suggesting diverse pathogenetic mechanisms [8]. Recent studies have identified somatic activating variants in the PDGFRB gene in fusiform cerebral aneurysms, implicating these variants in the etiology of the condition [9]. Nevertheless, the relationship between critical genetic determinants and the key pathological phenotype of intracranial fusiform aneurysm development remains unclear. Furthermore, the absence of conclusive evidence that the PDGFRβ activating variant directly results in fusiform aneurysms in animal models constitutes a significant obstacle for testing novel therapies in preclinical settings.

In this study, we utilized single-cell sequencing and immunofluorescence to uncover phenotype modulation of smooth muscle cells (SMCs) in fusiform intracranial aneurysms (FIAs), subsequently elucidating the underlying genetic factors contributing to these processes by confirming the presence of the PDGFRβ activating variant in our patient cohort. Finally, we endeavored to establish zebrafish models of mutated PDGFRB induced intracranial fusiform-like vascular anomalies to further our understanding of this condition.

## Materials and methods

### Clinical diagnosis of intracranial fusiform aneurysm

According to the relevant criteria, intracranial fusiform aneurysm patients were clinically diagnosed through a combination of medical history assessments and neuroradiological examinations, which included cerebral digital subtraction angiography (DSA) and computed tomography angiography (CTA) and/or magnetic resonance imaging (MRI) [2, 5]. Clinical and imaging data were sourced from medical records and Picture Archiving and Communication Systems, respectively.

### Intracranial fusiform aneurysm cell suspension preparation

Fresh intracranial fusiform aneurysm tissue was cut into approximately 1 mm^3^ pieces and dissociated using collagenase type I (#C2674, Sigma-Aldrich) for 10 min at 37 °C. Samples were then washed with Dulbecco's Modified Eagle's Medium (DMEM, #D5796, Sigma-Aldrich) and centrifuged (4 min at 300 g, 18 °C, minimal braking). Following this, samples were filtered through a 40 µm cell strainer with DPBS and washed with red blood cell lysis buffer (#555,899, BD Biosciences). The dissociated cell suspension was washed once and resuspended with DPBS.

### 10X Genomics single-cell RNA-Seq

Cell suspension from one intracranial fusiform aneurysm sample (AN-08) underwent single-cell RNA sequencing. This process was conducted using the 10X Genomics single-cell 3' library platform. The intracranial fusiform aneurysm cell suspension was loaded into Chromium microfluidic chips with 3' v3 chemistry and barcoded using a 10 × Chromium Controller (10X Genomics). All subsequent steps adhered to the standard manufacturer's protocols. Sequencing was performed with Illumina (NovaSeq) according to the manufacturer's instructions.

### Quality control and data processing of single-cell RNA-Seq

Using the Cell Ranger (v.3.0.2) Pipeline coupled with human reference version GRCh38, we generated raw gene expression matrices for each sample. Then, we analyzed the output filtered gene expression matrices using R software (v.4.2.2) with the Seurat43 package (v.3.0.0). Genes expressed at a proportion > 0.1% of the data and cells with > 200 genes detected were selected for further analyses, while low-quality cells were removed based on criteria such as < 800 unique molecular identifiers (UMIs), < 200 genes, or > 10% UMIs derived from the mitochondrial genome. After quality control, we normalized gene expression matrices using the NormalizeData function and identified 2,000 features with high cell-to-cell variation using the FindVariableFeatures function. To reduce dataset dimensionality, we applied the RunPCA function with default parameters to linear-transformation scaled data generated by the ScaleData function. Finally, we clustered cells using the FindNeighbors and FindClusters functions, and performed nonlinear dimensional reduction with the RunTSNE function using default settings.

### Sample preparation for whole-exome sequencing (WES)

For intracranial aneurysm specimens, sample tissue was placed in liquid nitrogen within five minutes post-resection, and subsequently stored at -80 °C as frozen samples for further whole-exome sequencing (WES). When feasible, peripheral blood samples were collected from intracranial fusiform aneurysm patients. These blood samples were placed into ethylenediaminetetraacetic acid anticoagulation tubes and stored at -80 °C.

### WES of intracranial fusiform aneurysm tissue and peripheral blood samples

Genomic DNA from intracranial fusiform aneurysm samples and peripheral blood was isolated using commercially available kits (QIAGEN Gentra Puregene and QIAamp DNA Blood Mini Kit) following established protocols. The quality of the isolated genomic DNA was assessed through a combination of two methods: (1) monitoring DNA degradation and contamination on 1% agarose gels and (2) measuring DNA concentration with a Qubit® DNA Assay Kit in a Qubit® 2.0 Fluorometer (Invitrogen, USA). For library preparation, a total of 0.6 μg genomic DNA per sample was used as input material. Using the Agilent SureSelect Human All Exon V6 kit (Agilent Technologies, CA, USA) according to the manufacturer's recommendations, sequencing libraries were generated, and index codes were added to each sample. DNA fragmentation into 180–280 bp fragments was performed using a hydrodynamic shearing system (Covaris, Massachusetts, USA), and any remaining overhangs were converted into blunt ends through exonuclease/polymerase activities. DNA fragments with ligated adapter molecules on both ends were selectively enriched in a PCR reaction. Next, libraries were hybridized in the liquid phase with biotin-labeled probes and exons of genes were captured using magnetic beads with streptomycin. Index tags were added to the purified products using the AMPure XP system (Beckman Coulter, Beverly, USA). Clustering of the index-coded samples was conducted on a cBot Cluster Generation System using the Hiseq PE Cluster Kit (Illumina) according to the manufacturer's instructions. Following cluster generation, the DNA libraries were sequenced on an Illumina Hiseq platform, generating 150 bp paired-end reads.

### Mapping WES

Burrows-Wheeler Aligner (BWA) was used for mapping the whole exome sequencing data to UCSC hg19 human genome reference with default parameters. We then utilized Picard for marking duplicates and SAMtools for sorting the alignments by coordinates. The depths of 313,346 autosomal common SNP loci were calculated with the multicov function from bedtools, and the median coverages for fusiform aneurysm tissue, patient blood were estimated to be 285 × and 113x, respectively.

### Quality control of WES

In order to filter out potential contaminated reads that fully match the reference of other species than human, only 150 alignment reads with (1) > 50 matched bases and (2) ≤ 4 mismatched bases were kept using pysam. The resulting cleaned BAM files were used for downstream analysis.

### Somatic mutation detection in WES

We used SAVI2 on 7 bulk fusiform aneurysm samples with matched blood for detecting somatic mutations. in three steps. Step 1, candidate variants were selected according to the following criteria: (1) read depth for paired blood and fusiform aneurysm ≥ 20, (2) paired blood VAF = 0%, (3) VAF ≥ 2% and number of altered reads in fusiform aneurysm ≥ 2, (4) not present in meganormal mutation database (defined by SAVI2), (5) not present in meganormal mutation database (defined by TCGA), (6) low multi-mapping possibilities (S1_alt_depth_per_position < 3), (7) no strand bias in fusiform aneurysm tissue (specifying in tissue: p-value for strand bias ≥ 0.05, alt_reverse_depth > 0 and alt_ forward_depth > 0), (8) non-common SNPs, (9) nonsynonymous mutations. Step 2, potential somatic mutations were prioritized by (1) a significant higher VAF in fusiform aneurysm tissue than in paired blood (savi_change_stat = ‘up’), (2) recurrent in more than one patient. Step 3, the prioritized somatic mutations were further manually checked via the Integrative Genomics Viewer (IGV) for clean surrounding area.

### Laser capture microdissection (LCM) and Sanger sequencing

LCM protocol was applied as previously described [10]. Sections obtained on polyethylene-naphthalate membrane glass slides (415,190–9081-000, Zeiss) were cut from FFPE tissue of one patient (AN-08) according to manufacturer’s instructions. Smooth muscle layer and endothelial layer were identified using the reference H&E and microdissected by using the microdissection system (PALM MicroBeamZeiss) and collected in the 0.2 ml adhesive cap tubes (415,190–9191-000, Zeiss). Genomic DNA was extracted using the QIAamp DNA Micro Kit (Qiagen, Hilden, Germany) according to the manufacturer’s protocol. The targeted region of PDGFRB (covering approximate 200 bp window around chr5 149,504,131) from SMCs and ECs was amplified using Taq master mix (TakaRa, Dalian, China) in Applied Biosystems 2720 Thermal cycler. The samples were then cleaned up by EDTA and analyzed in a 3730 DNA Analyzer (3730-XL, Thermo Fisher). The remaining AN-09 samples after single-cell sequencing were embedded in paraffin and subjected to Sanger sequencing by the same method. The results of Sanger sequencing were shown in Supplementary Fig. 5.

### Adenovirus preparation

The adenovirus carrying PDGFRB mutations (Y562D, RYEIR561R, EI563, N666K) were designed, constructed, and produced by Beijing Hecheng Biotechnology Co. LTD (Beijing, China). Briefly, Adenoviruses carrying PDGFRB mutations were synthesized and cloned into pAD-CMV-MCS-IRES-EGFP vector. Then, the adenovirus was propagated in Ad293 cells and purified using the freeze–thaw method followed by CsCl density gradient centrifugation. The pAD-CMV-MCS-IRES-EGFP vectors, which express PDGFRB, were used as the Wild Type, while those expressing only EGFP were used as the Control. At the time of infection, plates were incubated with 20 MOI of adenoviral in culture medium.

### Cell culture and treatment of vascular smooth muscle cells (VSMCs)

Primary human brain vascular smooth muscle cells (HBVSMCs) were acquired from ScienCell and cultured in smooth muscle cell medium (SMCM, ScienCell) supplemented with 2% smooth muscle cell growth supplement (SMCGS, ScienCell) and 5% fetal bovine serum (FBS, ScienCell). Cells were maintained in a humidified atmosphere at 37℃ with 5% CO2.

Constructed PDGFRB adenovirus vectors (Control, Wild Type and Y562D vector) was transfected according to the manufacturer’s protocol. The complete medium was replaced after 24 h of transfection.

JAK inhibitor (Ruxolitinib, 100 nM, Selleck) was directly added to the complete medium. The medium containing the agents was replaced daily to ensure continuous drug action. All drugs were dissolved in DMSO, with comparisons made to vehicle-treated controls (i.e., DMSO, 0.1%).

### Starvation treatment of vascular smooth muscle cells (VSMCs)

Post adenovirus infection, HBVSMCs were starved for 48 h and stimulated with 25 and 100ηg/mL of Human Platelet-Derived Growth Factor BB (hPDGF-BB) (#50,611, CST) for 30 min.

### Western blot

At 48 h post adenovirus infection, whole-cell lysates were prepared utilizing RIPA buffer (Sigma-Aldrich, St. Louis, USA). Protein concentrations were determined via a BCA Protein Assay kit (Pierce). Equal amounts of total proteins (20 µg) were loaded onto gels, separated through SDS-PAGE, and transferred to polyvinylidene difluoride membranes (PVDF) (Merck KGaA, Darmstadt, Germany). Signals were detected using the Immobilon Western Chemiluminescent HRP Substrate (WBKLS0500, Millipore, MA). Glyceraldehyde 3-phosphate dehydrogenase (GAPDH) served as the loading control. The primary antibodies employed included: anti-p-JAK2 (1:1000, #3771, CST), anti-p-STAT1 (1:1000, #9167, CST), anti-p-STAT3 (1:1000, #9145, CST), anti-PDGFRB (1:1000, #3169, CST), anti-α-SMA (1:1000, #19,245, CST), anti-SM22α (1:1000, ab14106, Abcam), anti-MMP1 (1:1000, ab137332, Abcam), anti-MMP9 (1:1000, ab76003, Abcam), anti-VCAM1 (1:1000, ab134047, Abcam), anti-ICAM1 (1:1000, ab109361, Abcam), anti-GAPDH (1:2500, ab9485, Abcam), anti-p-Src (1:1000, #6943, CST), anti-p-PLCγ (1:1000, #14,008, CST), anti-p-Erk1/2 (1:1000, #4370, CST).

### Immunoprecipitation

Immunoprecipitation (IP) was performed with Protein A/G Immunoprecipitation Kit (MT2400, Solarbio). HBVSMCs transfected with different mutant viruses (RYEIR561R, EI563, N666K, Y562D) were cultured for 48 h and whole-cell lysates were extracted following the manufacturer’s protocol. IP’d receptors were western blotted using anti-phospho-Tyrosine Mouse mAb (1:1000, sc-7020) from Santa Curz.

### RNA isolation and real time quantitative (RT-qPCR)

Total RNA was isolated using the TRIzol method. RNA purity and concentration were evaluated using a NanoPhotometer® spectrophotometer (IMPLEN, Germany) and measured with a Qubit® RNA Assay Kit in conjunction with a Qubit® 2.0 Fluorometer (Life Technologies, USA). RNA integrity was assessed using the RNA Nano 6000 Assay Kit in a Bioanalyzer 2100 system (Agilent Technologies, USA). RNA was purified with gDNA Eraser (Takara, Kyoto, Japan). After determining RNA concentration, purified RNA was reverse-transcribed employing a PrimeScript™ RT reagent Kit (Takara). qPCR was conducted using TB Green Premix Ex Taq (Takara) and a QuantStudio™ 3 System (Applied Biosystems) alongside specific primers. GAPDH was utilized as a reference gene. The primer sequences are provided in Supplementary Table 2.

### Immunofluorescence staining

For polychromatic immunofluorescence, the Opal Multiplex IHC Assay Kit (PerkinElmer, USA) was employed on formalin-fixed paraffin-embedded FIA sections following the manufacturer's instructions. Fluorescent images were acquired using a Zeiss Axio Scope A1 microscope. Primary antibodies included: anti-MMP-9 (1:20, BM4089, Boster), anti-α-SMA (1:20, BM3902, Boster), anti-ICAM1(1:50, A00171, Boster), anti-p-JAK2 (1:500, bs-3206R, Bioss), anti-p-Src (1:50, bs-1730R, Bioss), anti-p-PLCγ (1:100, #MAB74542, R&D systems), anti-p-Erk1/2 (1:100, bs-3016R, Bioss), anti-p-STAT1 (1:50, #7649, CST), anti-p-STAT3 (1:20, BM4835, Boster). Rabbit IgG (#3900, CST) or mouse IgG1 (#5415, CST) with the same concentration as primary antibodies were utilized as isotype controls to validate antibody specificity. Meanwhile, secondary antibody only controls were used to distinguish genuine target staining from background.

### Wound healing analysis

Cell motility was assessed using a scratch wound-healing assay. Wound healing assays were performed with an Ibidi Culture-Insert (Ibidi). VSMCs with different treatments were suspended in complete medium at a concentration of 700,000 cells/ml, and 70 µl of cell suspension was pipetted into each chamber of the cell culture insert. When the cell density reached more than 90% confluence, the culture insert was gently removed using sterile tweezers. Meanwhile, SMCM was replaced with serum-free medium. Images were acquired using an inverted microscope (IX51, OLYMPUS, Japan) at 0 h and 4 h after scratch. The percentage of the reduced area was measured using NIH ImageJ software (version 1.52a) and reported as a percentage.

### Cell proliferation analysis

5-Ethynyl-2′-deoxyuridine (EdU) assay was performed with an EdU Kit (C0078S, Beyotime) according to manufacturer’s instructions. Briefly, VSMCs with different treatments were seeded onto 12-well plates and cultured in SMCM medium for 48 h. Then switched into fresh SMCM medium supplemented with EdU (10 μmol/L) and incubated for 2 h. Cells were fixed with 4% paraformaldehyde for 15 min and permeated with 0.3% Triton X-100 (T8200, Solarbio) for another 15 min. Click reaction solution configured according to the instructions was added to each well. After 1 h of incubation in the dark, nuclei were stained with DAPI (C0060, Solarbio) for 10 min. Images were acquired with Invitrogen Evos FL Auto2 microscope (Invitrogen, USA). The proportion of EdU-positive cells was by measured using NIH ImageJ software (version 1.52a).

### Zebrafish strain maintenance

The zebrafish strain Tg(kdrl:eGFP) [11], AB, was used. All experiments involving zebrafish were approved by the South China University of Technology Animal Ethics Committee. According to previous research, zebrafish were raised under standard conditions with a 10-h dark, 14-h light cycle and at 28.5 °C [12]. All work involving zebrafish and mice was reviewed and approved by Animal Ethics Committee of South China University of Technology.

### Y562D/WT human PDGFRB mRNA synthesis and treatment in zebrafish

Using the PDGFRB pcDNA3.1-HA-C plasmid (YouBio, Changsha Zeqiong Biotech) as a template, the Y562D PDGFRB plasmid was amplified by PCR with a previously designed Y562D point-mutated primer pair. WT/Y562D Human PDGFRB mRNA was transcribed in vitro from the WT/Y562D PDGFRB pcDNA3.1-HA-C plasmid using the mMESSAGE mMACHINE™ T7 in vitro Transcription Kit (AM1344, Invitrogen). WT/Y562D Human PDGFRB mRNA (500 ng/μl) was injected into a single zygote cell. The embryos were immersed in egg water containing 800 nM Ruxolitinib from 24 h post-fertilization to 3 days post-fertilization.

### Immunofluorescence for zebra fish

To examine the spatial patterns of vessels and erythrocytes, embryos were stained using goat anti-GFP (1:400 dilution, Abcam) and rabbit anti-αe1 (1:400 dilution, donated by Professor Wen's lab, The Hong Kong University of Science and Technology). GFP staining was visualized with Alexa Fluor donkey anti-goat-488 and Alexa Fluor donkey anti-goat-555 (1:400, Invitrogen). Images of zebrafish immunofluorescent staining were captured using a Zeiss LSM800 laser scanning confocal microscope in dual laser channel mode, and the relative positions of vessels and red blood cells were observed. Oxford BC43 was used for volumetric imaging of the head vasculature and microglia in zebrafish. Statistical analysis of the number of glial cells around and within blood vessels.

### Neurobehavioral performance

5dpf zebrafish were placed in a 24-well plate and placed in DanioVision instrument for three rounds of bright (10 min) -dark (10 min) treatment, and their movements were recorded at the same time.

### Statistical analysis

GraphPad Prism V.8.00 and R V.3.0 were used for statistical analysis. For continuous variables that satisfy the assumptions of normal distribution and homogeneity of variance, the Student's t-test is used for two-group comparisons and Benjamini–Hochberg correction is employed for multiple-group comparisons. For continuous variables that do not conform to the normal distribution assumption, the Mann–Whitney U test is used for two-group comparisons, and the Dunn's test is utilized for multiple-group comparisons.

## Results

### Patients

In this study, we included nine intracranial aneurysm patients treated between January 2018 and March 2024, all of whom had fresh-frozen aneurysmal tissue and matched blood samples. For controls, cerebral arteries were obtained from two patients with traumatic brain injury and two patients who underwent temporal lobe resection for epilepsy during standard neurosurgical procedures. Clinical information, sample details and preoperative imaging of the intracranial aneurysm patients can be found in Supplementary Table 1 and Supplementary Fig. 1.

The patient ages ranged from 24 to 68 years, with a mean age of 45.33 years. Among these patients, four were male and five were female. Three patients had hypertension; one patient had hyperlipidemia. One patient presented with subarachnoid hemorrhage (SAH); one with intracranial hematoma (ICH); one with dizziness; one with ischemic neurological deficits due to massive cerebral infarction; four with headache and one with trauma. Five aneurysms were located in the middle cerebral artery, while three were in the anterior cerebral artery, the remaining one was located in anterior communicating artery. The etiology of the aneurysms varied, one cases may be attributed to trauma, one to dissection and the remaining cases were unknown.

### Smooth muscle cell phenotype modulation in intracranial fusiform aneurysm

**Fig. 1:**
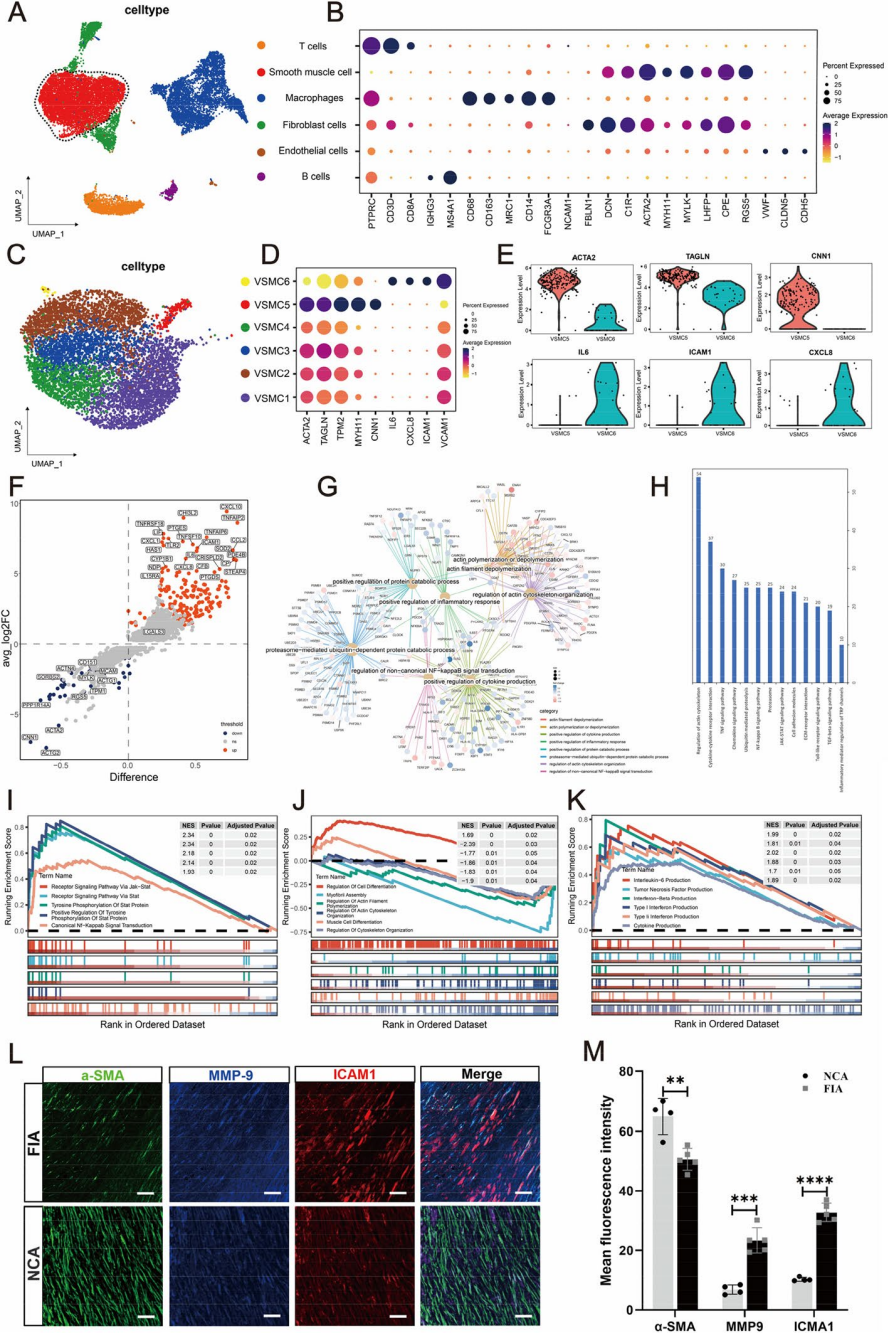
Single-cell transcriptional profiling of intracranial fusiform aneurysmal cells (**A**-**K**) and multi-color immunofluorescence (mIF) of smooth muscle cells (SMCs) markers and inflammatory markers between intracranial fusiform aneurysms (IFAs) and normal cerebral arteries (NCAs) (**L**-**M**). T-SNE visualization of intracranial fusiform aneurysmal cells type. Colored according to cell type (**A**). Visualization of specific gene expression patterns related to cell subsets identified in (**A**) using a bubble plot (**B**). T-SNE visualization of VSMC cell clusters. Colored according to clusters (**C**). Visualization of structural protein and inflammation-related genes expression patterns within the subsets of smooth muscle cells identified in (**C**) using a bubble plot (**D**). The violin plots show the expression differences of structural protein genes and inflammatory genes between the contraction subgroup (VSMC5) and the inflammatory subgroup (VSMC6) (**E**). The volcano plot specifically shows the gene expression differences between the two cell groups (**F**). The petal plot displays the GO enrichment analysis results of differential genes between the VSMC5 and VSMC6 (**G**). The bar graph shows the KEGG enrichment analysis results of differential genes between these two cell subsets (**H**). The GSEA enrichment analysis results of differential genes between the two groups. The enrichment results of differential genes related to signaling pathways (**I**). The enrichment results of differential genes related to structural protein genes (**J**). The enrichment results of differential genes related to the process of inflammatory factor secretion (**K**). α-SMA (green, SMCs marker), MMP-9 (blue, inflammatory marker) and ICAM-1 (red, inflammatory marker) in FIAs and NCAs are detected using mIF. Scale bar, 100 μm (**L**). Statistical analysis of mean fluorescence intensity about SMCs marker (α-SMA) and inflammatory markers (ICAM-1 and MMP-9) between NCAs (*n* = 4) and FIAs (*n* = 5). 'n' represented the number of samples. Three random fields were selected for statistical analysis in each sample, and the average value represented the detection value of this marker in this sample. The Student's t-test is utilized to examine the statistical differences among each marker. ns, no significant; **p < 0.01, ***p < 0.01 (M). n values indicate number of independent experiments performed

AN-08 (Y562D) and AN-09 (Y562C) with PDGFRB mutations identified by Sanger sequencing (Supplementary Fig. 5) were used for single-cell sequencing to investigate the composition and changes of VSMCs in intracranial fusiform aneurysms at the single-cell level. We used the “singleR” (R package) to identify the cells into six subgroups (T cells, Vascular smooth muscle cells, Macrophages, Fibroblast cells, Endothelial cells, and B cells) (Fig. 1A and B). Subsequently, we extracted VSMCs and detected six distinct VSMCs clusters (VSMC1, VSMC2, VSMC3, VSMC4, VSMC5 and VSMC6) (Fig. 1C), according to the expression of genes. The gene profiles of these subsets demonstrated distinct differences (Fig. 1D). Among them, SMC5 primarily expressed VSMCs nmarker genes (ACTA2, TAGLN, TPM2, MYH11, and CNN1), consistent with a typical contractile phenotype. In contrast, SMC6 showed decreased expression these marker genes, accompanied by high expression of inflammatory genes (IL6, CXCL8, ICAM1, and VCAM1), consistent with an inflammatory phenotype. The remaining subsets fall somewhere in between, resembling a subset with transitional state (Fig. 1D). The VSMCs can change their phenotype with different characteristics and behavior in response to various stimuli or pathological conditions [13–15]. The heterogeneous state of VSMCs in FIAs suggested a possible phenotypic shift. To further explore the gene expression during inflammatory transformation, we compared the differences in gene expression between the typical contractile phenotype subset (VSMC5) and the inflammatory phenotype subset (VSMC6). The results of differential gene analysis showed that a large number of inflammation-related genes were up-regulated in VSMC6, accompanied by a decrease in the expression of smooth muscle structural protein genes (Fig. 1E and F). To further explore the biological processes and signaling pathways represented by these differentially expressed genes, GO, KEGG and GSEA analyses were performed. GO analysis revealed that differentially expressed genes were mainly involved in smooth muscle structure protein regulation processes (actin polymerization or depolymerization, actin filament depolymerization, regulation of actin cytoskeleton organization) and inflammation regulation processes (regulation of non-canonical NF-kappaB signal transduction, positive regulation of cytokine production, and positive regulation of inflammatory response) (Fig. 1G). In addition to these findings, KEGG enrichment analysis also revealed that differentially expressed genes were mainly involved in the JAK-STAT signaling pathway and ECM-receptor interaction pathway (Fig. 1H). Further GSEA analysis clarified that the upregulated genes in the inflammatory transformation subset (VSMCs6) mainly focused on cell inflammation regulation pathways (such as Receptor Signaling Pathway Via Jak-Stat and Canonical Nf-Kappab Signal Transduction) (Fig. 1I). Furthermore, the upregulated genes were also enriched in processes related to the secretion of inflammatory factors (such as Interleukin-6 Production, Tumor Necrosis Factor Production, Interferon-Beta Production, and Type I Interferon Production) (Fig. 1 K). In contrast to the typical contractile phenotype, the downregulated genes were mainly enriched in processes related to the Regulation Of Cytoskeleton Organization and Myofibril Assembly Regulation Of Actin (Fig. 1 J).

To validate the SMC phenotype modulation hypothesis, we performed multicolor immunofluorescent staining (mIF) to examine marker proteins associated with phenotypic modulation (including α-SMA, MMP-9 and ICAM1) in fusiform aneurysm and cerebral arteries (Fig. 1L). Significantly, inflammatory-related markers, such as MMP-9 and ICAM1, exhibited prominent staining within smooth muscle layer of fusiform aneurysm lesions, whereas their expression was scarce in cerebral arteries. Meanwhile, the smooth muscle cell marker α-SMA exhibited weak staining within smooth muscle layer of fusiform aneurysms compared to cerebral arteries (Fig. 1 M).

Overall, The results of scRNA-seq and mIF suggested that there was clearly an inflammatory phenotypic transformation in intracranial fusiform aneurysmal VSMCs.

### PDGFRB somatic mutation in smooth muscle cells in fusiform aneurysm

**Fig. 2:**
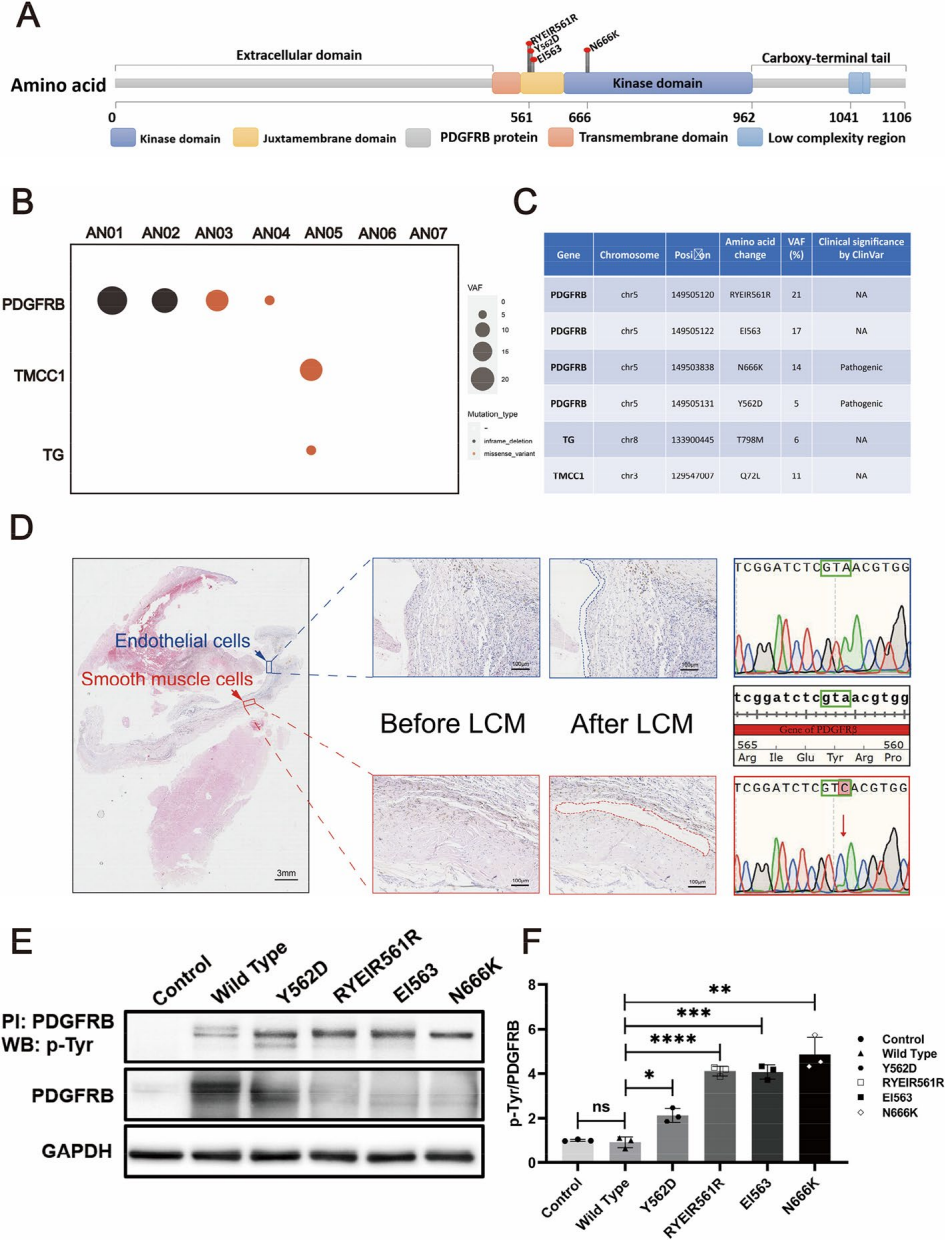
Somatic mutational landscape of FIAs. Structure of the protein encoded by PDGFRβ and the lollipop plots illustrate the location of the identified mutations in our cohort. The color of the boxes denotes different regions of the encoded protein. The coordinates represent the n-th amino acids (**A**). Somatic mutational landscape of 7 FIA samples with paired blood. The x-axis and y-axis specify the gene name and sample ID, respectively. The size of the circle represents the variant allele frequency. And the color denotes different types of mutations (**B**). Clinical significance of the detected somatic mutations according to the ClinVar database (**C**). The smooth muscle layer and endothelial layer in the FIA sections are dissected by laser-capture microdissection (LCM). PDGFRB.^Y562D^ mutation is found in smooth muscle layer by Sanger sequencing (**D**). HBVSMCs with PDGFRB mutations identified by WES are constructed and these mutations are found to have a characteristic with gain-of-function by immunoprecipitation (**E**). The scatter bar graph is shown the relative density of immunoblot bands (normalized to those in cells transfected vector) about pTyr/PDGFRβ in cells expressing different PDGFRB mutations is shown in (**F**) (*n* = 3). ‘n’ represents three repeated experiments. The Student's t-test was employed to assess the differences in pTyr/PDGFRβ levels between different mutant groups and the Wild Type group, with Bonferroni correction for multiple comparisons. n values represent the number of repeated experiments. ns, no significant, **padj < 0.01, ***padj < 0.001

Whole-exome sequencing was performed on tissue samples of cerebral aneurysm obtained from seven patients (six fusiform and one saccular). Using the default settings of SAVI2 and mutation prioritization criteria (Supplementary Fig. 2), we detected five somatic variants in three genes, including three missense mutations and two inframe deletions (Fig. 2B). Interestingly, three of the identified variants were located on the gene PDGFRB including two inframe deletion mutations and one missense mutation (chr5: 149,505,122, chr5: 149,505,120, chr5: 149,503,838), which corresponded to a c.1687_1692del (p.Glu563_Ile564del) mutation (in Patient AN-02), a c.1683_1694del (p.Tyr562_Arg565del) mutation (in Patient AN-01), and a c.1998G → C (p. Asn666Lys) mutation (in Patient AN-03). These variants had a variant allele frequency ranging from 17 to 21% (mean sequence coverage 357 ×). Focusing on variations on PDGFRB, another somatic mutation was detected (chr5: 149,505,131, c.1684A → C, p. Tyr562Asp) in Patient AN-04 with a relatively low variant allele frequency of 5% (sequence coverage 492x). Altogether, four somatic mutations were detected on gene PDGFRB in four/seven patients in our cohort. Details regarding the four mutations within the PDGFR regions can be found in Supplementary Fig. 3. On analyzing the seven paired blood samples, the exomes had good coverage for these site (mean sequence coverage ranging from 91 to 270 ×) and there are no sequence reads containing the same variants. The amino acids affected by the two inframe deletions (c.1688_1693delGATCTC, c.1682_1693delCGGATCTCGTAA) and one missense mutations (c.1684A → C) were located close to each other in the juxtamembrane region of the protein and by the other missense mutation (c.1998G → C) was located on the tyrosine kinase region (Fig. 2A). In terms of clinical significance of the two missense mutations on PDGFRB, namely c.1998G → C and c.1684A → C, was annotated as pathogenic in the ClinVar database (Fig. 2C). And they were previously reported to be gain-of-function mutations associated with sporadic infantile and pediatric myofibromatosis [16, 17]. In addition, the first report of somatic mutations in fusiform cerebral aneurysms detected a single novel variant within PDGFRB (p.Tyr562Cys [c.1685A > G]) in one patient. The reported variant led to the alteration of the same protein influenced by one of the missense mutations detected in out cohort (p. Tyr562Asp [c.1684A → C]), suggesting the importance of the integrity of the protein juxtamemebrane region [9]. We also detected two other somatic missense mutations on genes TG and TMCC1 in Patient AN-05 with variant allele frequencies of 6% and 11%, respectively. The former is a glycoprotein homodimer associated with thyroid function and the latter is an endoplasmic reticulum membrane protein.

We also conducted further experiments to pinpoint these mutations to specific cell types. A residual fusiform aneurysm sample (AN-08) in paraffin section was utilized for laser microdissection to separately extract endothelial layer and smooth muscle layer for Sanger sequencing. We identified the mutations (Y562D) in the smooth muscle layer, whereas no corresponding mutations in the PDGFRB gene were detected in the endothelial layer (Fig. 2D).

To further investigate the effect of these variants on PDGFRβ function, adenovirus-infected cell lines with PDGFRB mutations found in WES (RYEIR561R, EI563, N666K, Y562D) were constructed. The subsequent immunoprecipitation experiments unveiled a higher level of phosphorylated PDGFRβ in these mutant groups when compared to the Control and Wild Type groups, providing support for the gain-of-function role of these mutations on PDGFRβ function (Fig. 2E and F).

In summary, these gain-of-function PDGFRB mutations in the VSMCs may represent significant contributing factors to the development of fusiform aneurysms.

### PDGFRB somatic mutation induced smooth muscle cells phenotype modulation in vitro

**Fig. 3:**
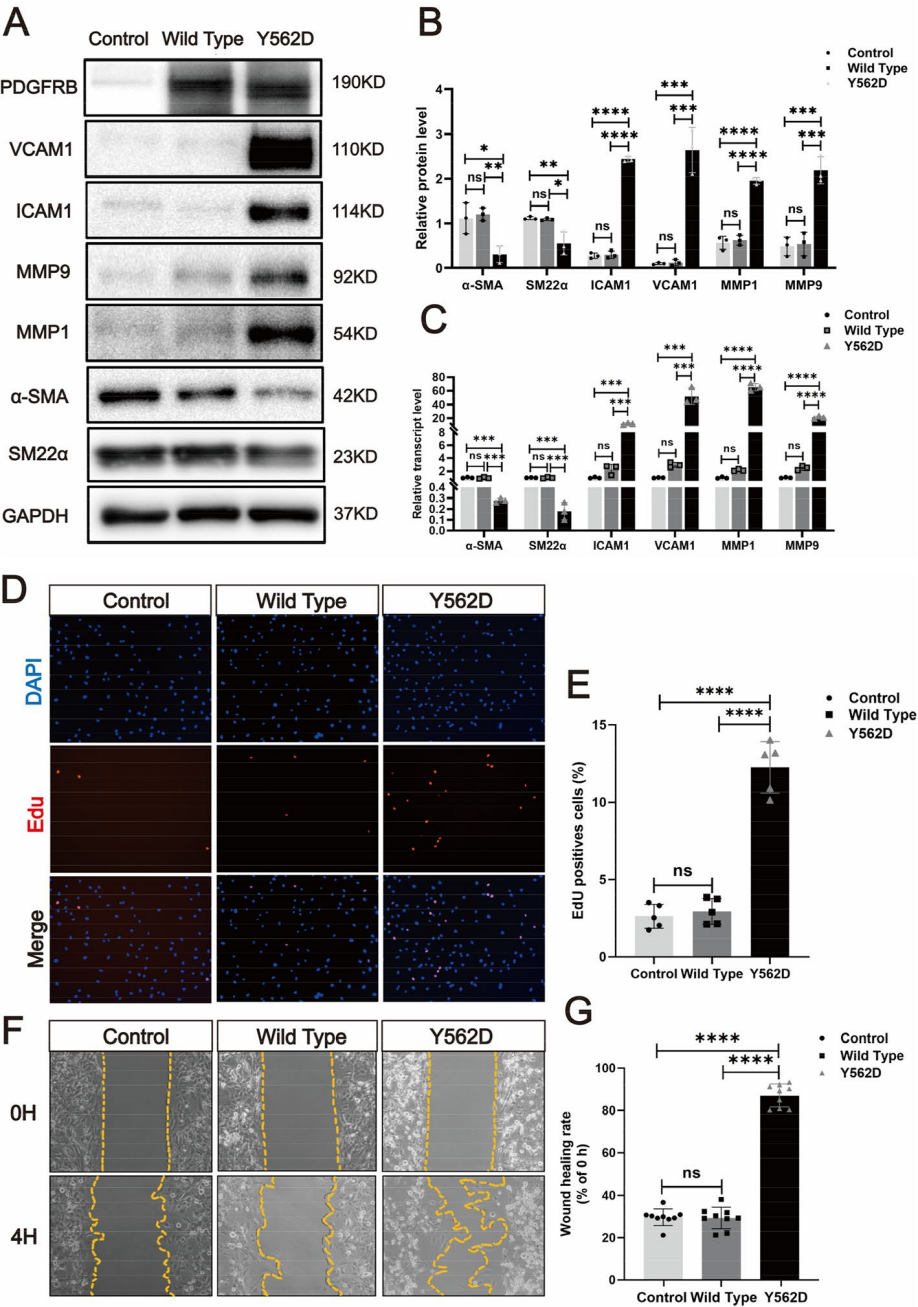
PDGFRB somatic mutation induce phenotypic modulation in SMCs. Immunostaining reveals the expression levels of smooth muscle markers (a-SMA and SM22a) and inflammatory markers (VCAM1, ICAM1, MMP1 and MMP9) in HBVSMCs transfected with different viruses (Control: vector; Wild Type: PDGFRB; Y562D: PDGFRB^Y562D^) (**a**). The relative density of immunoblot bands about markers shown in (**A**) were display (**B**) (normalized to those in cells transfected with vector viruses). RT-qPCR (**C**) of SMCs markers (α-SMA and SM22α) and inflammatory markers (VCAM-1, ICAM1, MMP-9 and MMP-1) in HBVSMCs underwent different treatments. Student's t-test and Benjamini–Hochberg correction are employed to assess the statistical significance. Edu assay exhibit the proliferation ability of HBVSMCs under different treatment conditions (**D**). Statistical analysis of the proportion of Edu-positive cells in the different groups from 5 different fields of each group at × 200 magnification. Tukey's multiple comparisons test is used for statistical differences (**E**). Scratch assay displays migratory ability of HBVSMCs underwent different treatment (**F**). Statistical analysis of the rate of wound healing (reduced area at 4H /area at 0H) in the different groups from 9 different fields of each group at × 200 magnification. Tukey's multiple comparisons test is utilized to evaluate the statistical significance. *padj < 0.05, **padj < 0.01, ***padj < 0.001, ****padj < 0.0001 (**G**). The above experiments are all repeated three times

To investigate the potential role of PDGFRB^Y562D^ in regulating VSMCs phenotype modulation, we generated human brain vascular smooth muscle cells (HBVSMCs) expressing PDGFRB^Y562D.^ We examined commonly used SMC markers (including α-SMA and SM22α) and inflammation-related markers (ICAM1, VCAM1, MMP1, MMP9). It observed upregulation of inflammation markers and suppression of SMC markers in PDGFRB^Y562D^-overexpressing human brain vascular smooth muscle cells (HBVSMCs) via Western blotting (Fig. 3A and B) and RT-qPCR (Fig. 3C). Additionally, PDGFRB^Y562D^-HBVSMCs exhibited enhanced proliferation ability (Fig. 3D and E) and migratory ability (Fig. 3F and G). These results collectively confirmed the role of activating PDGFRB^Y562D^ in driving phenotype modulation in vitro.

### JAK-STAT pathway paticipated in the SMCs phenotype modulation induced by PDGFRB somatic mutation

**Fig. 4:**
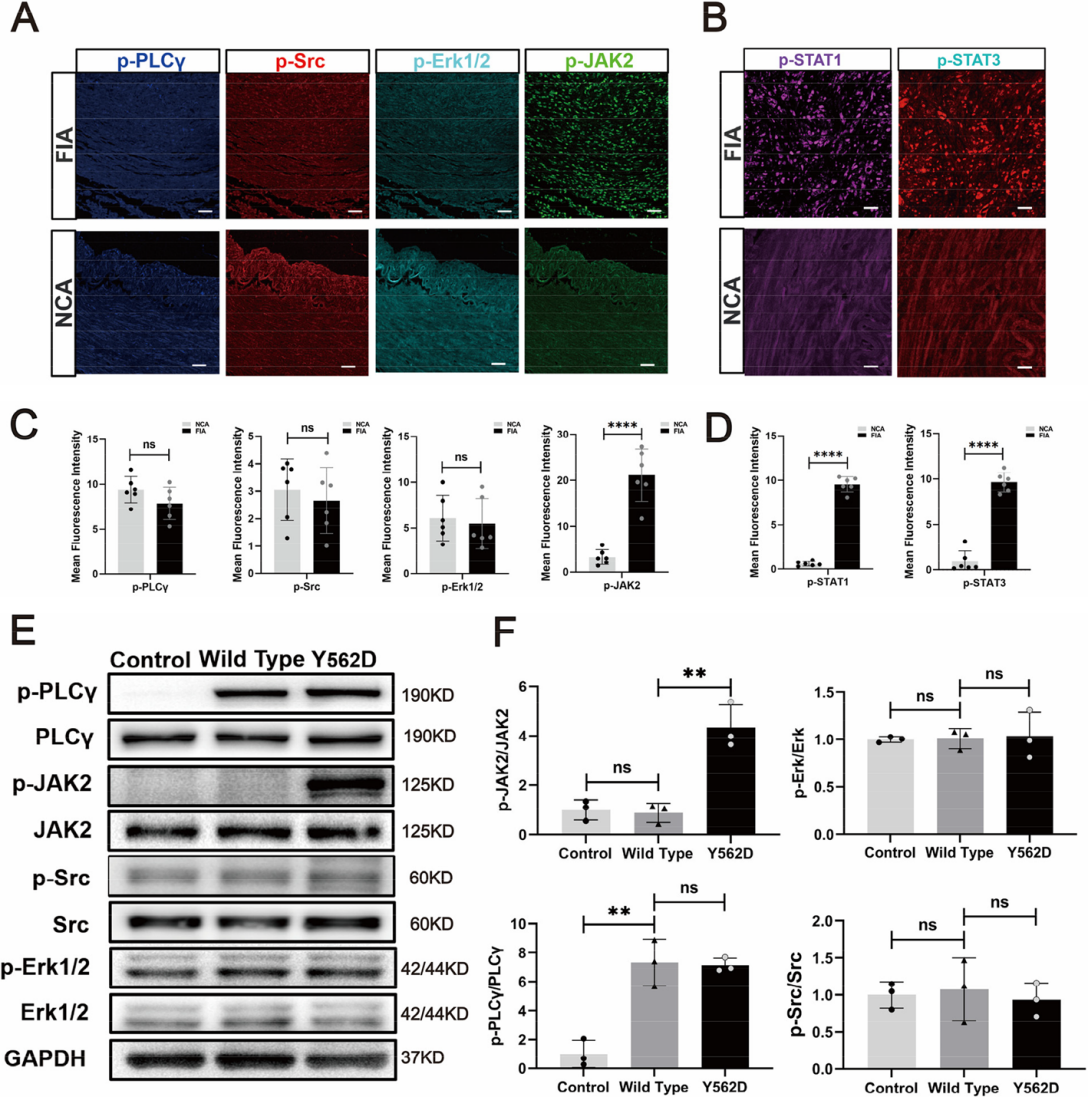
The JAK2-STAT pathway serves as the major downstream signaling pathway of the mutated PDGFB. mIF demonstrate the important downstream signaling pathway markers of PDGFRβ (p-JAK, p-Src, p-Erk1/2 and p-PLCγ) in FIA sections and NCA sections (**A**). Scale bar, 100 μm. Statistical analysis is performed to compare the mean fluorescence intensity of different signal markers between NCAs (*n* = 4) and FIAs (*n* = 5) (**C**). The expression of p-STAT1 and p-STAT3, which are downstream of p-JAK2, is detected by mIF in FIAs and NCAs. Scale bar, 50 μm (**B**). Statistical analysis of mean fluorescence intensity of these marks between NCAs (*n* = 4) and FIAs (*n* = 5) (**D**). Western blotting displays the expression of the important downstream signal pathway markers of PDGFRβ (p-JAK2, p-Src, p-Erk1/2 and p-PLCγ) in the HBVSMCs underwent different treatment in vitro (**E**). Relative density (normalized to Control) of immunoblot bands from (**E**) corresponding to pPLCγ, p-JAK2, p-Src, and p-ERK1/2 (*n* = 3) (**F**). 'n' represented the number of samples. Three random fields were selected for statistical analysis in each sample, and the average value represented the detection value of this marker in this sample. Tukey's multiple comparisons test is conducted to evaluate the statistical differences. ns, no significant; ***padj < 0.001, ****padj < 0.0001

The molecules JAK2, Src, PLCγ, and Erk1/2 are considered key components of the PDGFRβ downstream signaling pathways, and they are essential in activating PDGFRβ and regulating diverse cellular processes [18–21]. Immunofluorescence analysis showed that among the downstream signaling pathway markers of PDGFRβ, phosphorylated JAK2 (p-JAK2) was significantly elevated in fusiform aneurysms (Fig. 4A and C). Moreover, as the downstream targets of JAK2, phosphorylation of signal transducer and activator of transcription 3 and 1 (p-STAT1 and p-STAT3) were also significantly higher in fusiform aneurysms compared with the normal cerebral arteries (Fig. 4B and D). To further investigate the effect of the PDGFRβ (Y562D mutation) on downstream pathways, the levels of p-Erk1/2, p-Src, p-PLCγ, and p-JAK2 in HBVSMCs undergone different treatments were measured by Western blotting. The results indicated that the expression levels of p-PLCγ and p-Src, were found to be upregulated in both the Wild Type and Y562D groups. Notably, the expression of p-JAK2 was primarily observed at significantly higher levels in the mutant group, while there was no significant difference in the expression levels of p-PLCγ and p-Src between the Wild Type and Y562D groups (Fig. 4E and F). Subsequent serum deprivation experiments showed that the expression of p-JAK2 was still increased in the mutant group after excluding the interference of PDGFRBB in serum, suggesting that the increased expression of p-JAK2 was not affected by the serum PDGFBB (Supplementary Fig. 4). Collectively, these findings suggested that the p-JAK2 may play a major role in the downstream signaling of PDGFRB somatic mutation in fusiform aneurysms.

**Fig. 5:**
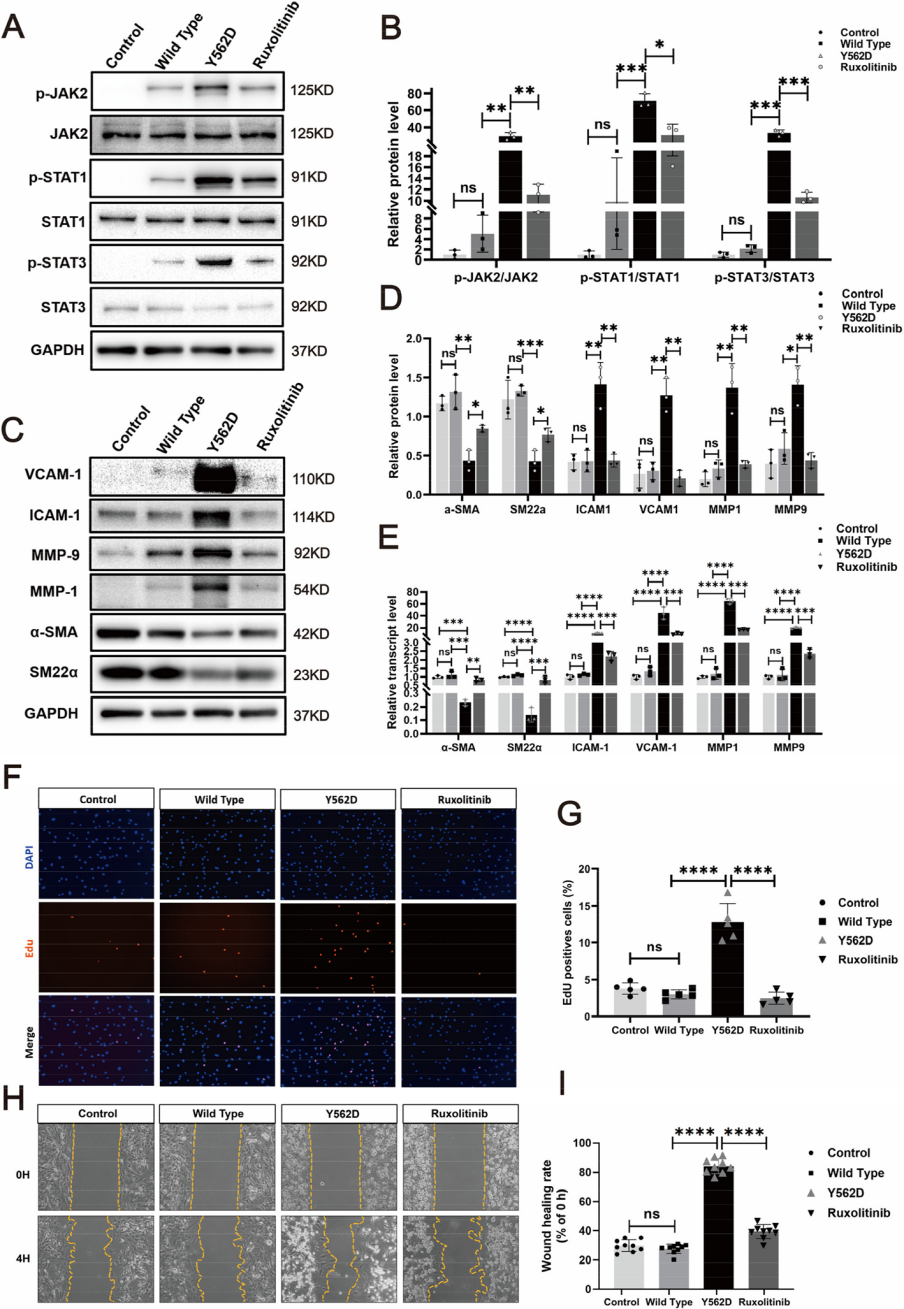
Ruxolitinib effectively reverse the PDGFRB^Y562D^-induced phenotypic modulation in HBVSMCs. Western blotting illustrates the alterations in p-JAK2 and p-STAT (p-STAT1 and p-STAT3) expression across different treatment groups (**A**). After normalization to the control group, the relative density of immunoblot bands in (**A**) is presented in scatter bar graph (**B**). Immunoblots (**C**) and relative density of bands (**D**) of markers associated with phenotypic modulation in HBVSMCs under different treatments are shown. A bar graph is used to present the rt-qPCR results for smooth muscle cell (SMC) markers and inflammatory markers in HBVSMCs under different treatment conditions (**E**). The results of RT-qPCR are analyzed using Tukey's multiple comparisons test. EDU assay demonstrate the proliferative capacity of HBVSMCs receiving different treatments (**F**) and statistical analysis of the proportion of Edu-positive cells in the different groups from 5 different fields of each group at × 200 magnification. Statistical differences are detected using Tukey's multiple comparisons test (**G**). Scratch assay shows the proliferative capacity of HBVSMCs underwent different treatment (**H**) and statistical analysis of the rate of wound healing (reduced area at 4H /area at 0H) in the different groups from 5 different fields of each group at × 200 magnification. Statistical differences are detected using Tukey's multiple comparisons test (**I**). ns, no significant; *p < 0.05; **p < 0.01. The above experiments are all repeated three times

We subsequently inhibited the JAK2-STAT pathway using the JAK-specific inhibitor Ruxolitinib. The Western blotting results demonstrated reduced expression levels of p-JAK2 and p-STAT in the PDGFRβ (Y562D mutation) -HBVSMCs following inhibitor treatment, indicating the efficacy of the inhibitors (Fig. 5A and B). Furthermore, both Western blotting targeting phenotypic transition markers in VSMCs revealed a decrease in inflammatory markers (ICAM1, VCAM1, MMP1 and MMP9) expression and a recovery of smooth muscle cell markers (α-SMA and SM22α) in PDGFRB (Y562D mutation) -HBVSMCs treated with inhibitors (Fig. 5C and D). Similar experimental findings were also observed through RT-qPCR (Fig. 5E). These findings suggested that inhibiting p-JAK2 effectively reversed the PDGFRB-driven inflammatory transformation, providing evidence for the significant involvement of the JAK2-STAT pathway in the phenotypic transition of HBVSMCs. Additionally, the inhibitor also attenuated the proliferation (Fig. 5F and G) and migration ability (Fig. 5H and I) of PDGFRB (Y562D mutation) -HBVSMCs. These findings provided evidence supporting the involvement of the JAK2-STAT pathway in mediating the transformation of smooth muscle phenotype driven by the PDGFRB mutations.

### PDGFRB somatic mutation induced fusiform aneurysm phenotype in vivo and were inhibited by JAK-STAT inhibition

**Fig. 6:**
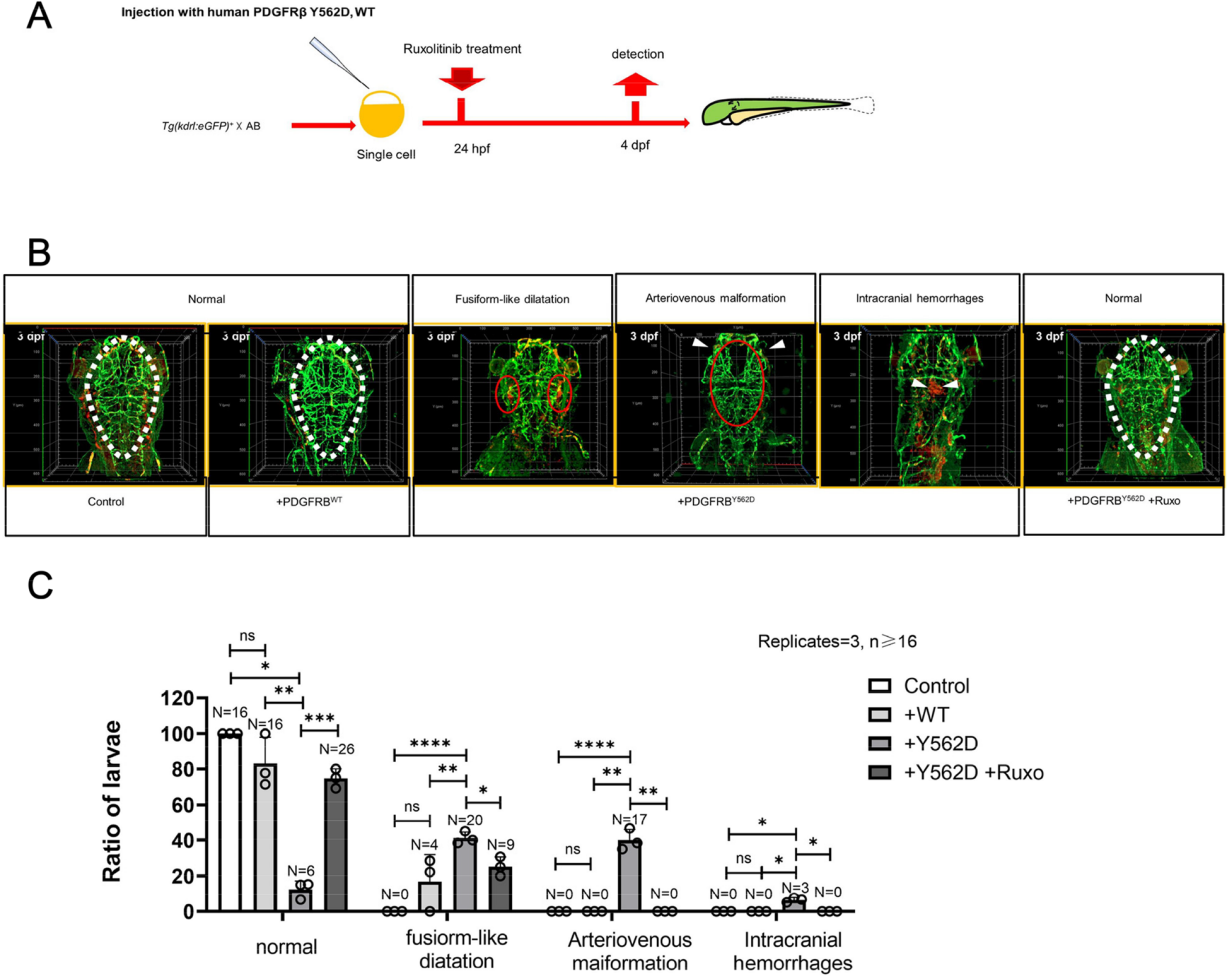
Ruxolitinib can reverse the phenotype caused by the PDGFRβ.^Y562D^ in zebrafish. **A** The timeline of human WT/Y562D PDGFRβ mRNA treatment and medication administration: Embryos are injected human WT/Y562D PDGFRβ mRNA at one-cell stage and treated with Ruxolitinib at 24 h post-fertilization (24 hpf) and preserve at 3 days post-fertilization (3 dpf). **B** and **C** Immunostaining of kdrl:eGFP + cells and erythrocytes(αe1) in the head of a 3 dpf embryos treated or not with Ruxolitinib. Statistical significance is determined using Student's t-test and Benjamini–Hochberg correction. ns, no significant; *padj < 0.05, **padj < 0.01, ***padj < 0.001, ****padj < 0.0001

To further investigate the impact of the human PDGFRB (Y562D) mutation on vascular development in vivo and the reversal effect of Ruxolitinib on the phenotype caused by Y562D, we employed Tg (kdrl:eGFP) zebrafish and conducted GFP and ae1 immunofluorescence staining (Fig. 6A and supplementary movie S1-6). The overexpression of human Y562D mutation could cause fusiform-like dilatation of the primordial hindbrain channel vessel wall (42 ± 3%), cerebrovascular malformations (39 ± 5%) and intracranial hemorrhages (7 ± 1%)(Fig. 6C). The results of the quantification analysis suggested that the abnormal phenotype displayed significant differences, particularly with regard to the morphology of aberrant central arteries [22]. No significant differences were observed between the PDGFRBWT group and the control group (Fig. 6C and supplementary movie S1 and S2). However, the + Y562D group displayed numerous abnormal intracranial angiogenesis individuals (approximately 88%) (Fig. 6B and supplementary movie S3-5). Furthermore, Oxford BC43 was used for volumetric imaging of the head vasculature and microglia in zebrafish (Supplementary Fig. 6). The results showed that PDGFRB Y562D-overexpressed zebrafish had more microglial infiltration around the blood vessels compared to wild-type zebrafish. This may suggest that the PDGFRB Y562D mutation could promote local vascular inflammation. However, no significant differences were observed in the neurobehavioral performance of zebrafish. This may be related to the location of the lesions and the short duration of observation (Supplementary Fig. 7). Interestingly, following treatment with Ruxolitinib, about 72% of the embryos recovered (Fig. 6B and supplementary movie S6). The Y562D mutation (+ Y562D group) could cause significant abnormalities in the development of cerebral vascular in zebrafish. In severe cases, it also resulted in cranial developmental defects, including the absence of eyes. Notably, Ruxolitinib treatment largely restored the phenotype induced by human PDGFRB^Y562D^. Consequently, Ruxolitinib has the potential to serve as a therapeutic agent for reversing the pathogenic effects of human PDGFRB ^Y562D^.

## Discussion

Fusiform aneurysms exhibit distinct underlying pathologies, hemodynamics, anatomical distributions, natural histories, and treatment approaches compared to saccular aneurysms, suggesting different mechanisms of pathological genesis [4, 8, 23]. Some somatic variants in the PDGFRB gene have been identified in these lesions [9]. Due to the challenge of obtaining fusiform aneurysm specimens, only a few studies have detected somatic mutations in fusiform aneurysms [9, 24]. In this study, we identified several PDGFRB gene variants, partially aligning with previous reports [9, 24]. Furthermore, no preclinical studies have tested whether these PDGFRB mutations are sufficient to drive the formation of abnormal cerebral vascular malformations. In our research, we observed fusiform-like cerebral vascular dilatation, cerebrovascular malformations and intracranial hemorrhages phenotypes in developing zebrafish transfected with PDGFRB mutations. The model demonstrated overt hemorrhage and altered vascular morphogenesis, confirming that activating PDGFRβ variants can drive the formation of fusiform-like vessel dilatation, or even more serious developmental abnormalities, in zebrafish.

The PDGFRB gene plays a role in the development and maintenance of blood vessels [20]. Mutations in this gene can cause a variety of vascular diseases, such as hereditary hemorrhagic telangiectasia (HHT) and chronic eosinophilic leukemia (CEL) [18, 25, 26]. Symptoms can range from nosebleeds and recurrent headaches to life-threatening conditions like heart failure and stroke [8, 27, 28]. Treatments may involve drugs targeting the mutated gene or surgical removal of blood vessels. These vascular diseases are primarily caused by loss-of-function mutations. In fusiform aneurysms, missense variants and in-frame deletions occur in either the ArgTyrGluIleArg motif of the juxtamembrane region or the adjacent AspPheGly motif in the activation loop of PDGFRβ. Disruption of juxtamembrane region auto-inhibitory sites results in constitutive activation [16, 29]. In this study, all four detected variants exhibited activation activity. Previous research localized the PDGFRB variant to CD31- (non-endothelial) cells in the aneurysmal radial artery but did not specify which cells contained the mutation [24]. In this study, using laser microdissection to obtain specific smooth muscle cell layer samples, we identified the PDGFRB variant within smooth muscle cells.

Based on histopathological findings of fusiform aneurysms, several pathological characteristics, including vessel dissection, atherosclerosis, and collagen dysplasia, have been observed in these lesions [30, 31]. Further investigation of pathological changes at the cellular and molecular levels is needed. In this study, we leveraged single-cell RNA sequencing technology to reveal phenotype changes from contractile to inflammatory status in smooth muscle cells harboring PDGFRB mutations. Furthermore, some cell signaling pathways related to inflammation have been identified. Among them, the NF-κb pathway has been shown to be associated with the activation of PDGFRB mutation in intracranial aneurysms [32]. Inflammation is considered a crucial pathological change in intracranial aneurysm, typically following endothelial dysfunction induced by hemodynamic stress [33]. We hypothesize that the initial evocation of an inflammatory response in fusiform aneurysms may be due to PDGFRB mutations, causing smooth muscle cell phenotype modulation characterized by decreased expression of contractile proteins and increased expression of inflammatory mediators and matrix metalloproteinases (MMPs), eventually leading to the aneurysm development.

The c.1684 site is located in the proximity membrane structure domain of PDGFRβ. Although the allele frequency in the population is low, it is the most frequently reported mutation site in the intracranial aneurysm [9, 32]. Self-activation may expose phosphorylation sites of tyrosine in the functional region of PDGFRβ, thereby creating docking sites for signaling proteins, including STAT transcription factors and phospholipase Cγ (PLCγ) ect [34–36]. The previous studies reported the JAK-STAT pathway played crucial roles in smooth muscle cell phenotype modulation [37–39]. In fusiform aneurysm specimens, we did observe JAK-STAT pathway were significantly activated. In addition, JAK-STAT pathway was also activated in the SMCs transfected with PDGFRB^Y562D^ mutations. Notably, using Ruxolitinib to inhibit the JAK-STAT pathway could significantly reduce the incidence of fusiform-like vascular dilatation and intracranial hemorrhages phenotypes induced by PDGFRB mutations, suggesting that this inhibitor was a promising option for targeted treatment of fusiform-like vascular dilatation such as fusiform aneurysms secondary to constitutive activation of PDGFRB^Y562D^ variants. Ruxolitinib has been used in clinical practice [40]. Besides common skin and gastrointestinal symptoms, high concentrations of Ruxolitinib can also cause impairment of bone marrow hematopoiesis [41]. Therefore, before conducting clinical trials, corresponding animal-level efficacy assessments are still needed.

There are several limitations and future directions for this research: First, the failure to demonstrate that PDGFRB activation in smooth muscle cells definitively leads to fusiform aneurysm formation in mammalian animal models remains a significant obstacle to testing the mechanisms driving fusiform aneurysm formation and other novel therapies in a preclinical setting. Second, whether other types of PDGFRB mutations share the same signaling mechanism is still unknown. Third, detecting these characteristics through radiological examinations would help identify suitable patients for targeted therapy. Finally, the targeted therapy, including Ruxolitinib, should be carefully evaluated in further studies on fusiform aneurysm treatment.

## Conclusion

Our study reveals that PDGFRB^Y562D^ somatic variants modulate vascular smooth muscle cell inflammatory transformation by activating the JAK-STAT pathway, implicating their involvement in the pathogenesis of FIAs. The effectiveness of Ruxolitinib, a JAK-STAT pathway inhibitor, demonstrated both in vitro and in vivo, suggests its potential as a therapeutic option for FIAs. Our findings underscore the promising therapeutic potential of targeting the JAK-STAT pathway for the treatment of FIAs, offering new avenues for clinical interventions in FIA patients.

## Limitation

Our study has several limitations. Firstly, due to the difficulty in obtaining samples of fusiform intracranial aneurysms, the number of single-cell samples included in the study was relatively small. This may have caused some interference with the analysis results. Secondly, we only investigated the common downstream cell pathways of PDGFRβ, and cannot determine whether mutations in PDGFRβ activate other pathways. Lastly, the limited number of samples included in the zebrafish neurobehavioral and immune cell infiltration studies may have interfered with the stability of the results. However, this finding is interesting, so we plan to further investigate it in future studies.

### Supplementary Information

Supplementary Material 1: Movie S1. Tg(kdrl:eGFP) control group, immunostaining of kdrl:eGFP+ cells and erythrocytes(αe1).

Supplementary Material 2: Movie S2.Tg(kdrl:eGFP) overexpression of human PDGFRBWTgroup, immunostaining of kdrl:eGFP+ cells and erythrocytes(αe1).

Supplementary Material 3: Movie S3.Tg(kdrl:eGFP) overexpression of human PDGFRBY562Dgroup showed Fusiform-like dilatation, immunostaining of kdrl:eGFP+ cells and erythrocytes(αe1).

Supplementary Material 4: Movie S4.Tg(kdrl:eGFP) overexpression of human PDGFRBY562Dgroup showed brain arteriovenous malformation, immunostaining of kdrl:eGFP+ cells and erythrocytes(αe1).

Supplementary Material 5: Movie S5.Tg(kdrl:eGFP) overexpression of human PDGFRBY562Dgroup showed Intracranial hemorrhages, immunostaining of kdrl:eGFP+ cells and erythrocytes(αe1).

Supplementary Material 6: Movie S6. Tg(kdrl:eGFP) overexpression of human PDGFRBY562D following treatment with Ruxolitinib group, immunostaining of kdrl:eGFP+ cells and erythrocytes(αe1).

Supplementary Material 7: Supplementary Figure 1: Imaging information of 9 patients with intracranial aneurysms (All fusiform aneurysms except AN-05). Supplementary Figure 2:The workflow of Whole-exome sequencing. Supplementary Figure 3: Details of the four mutations located within the PDGFRB gene region. Supplementary Figure 4:Starvation treatment and PDGFBB stimulation. Supplementary Figure 5:Sanger sequencing results of AN-08 and AN-09. Supplementary Figure 6:The infiltration of microglia around the head vasculature in zebrafish. The left side shows the statistical results of microglial cell counts around the blood vessels in zebrafish from each treatment group. The right side shows the infiltration of microglial cells around the head vasculature in zebrafish from each treatment group (red represents microglial cells, green represents blood vessels). Supplementary Figure 7:5-day post-fertilization (dpf) zebrafish locomotor activity: Left panel shows the distance traveled by zebrafish within one hour; Right panel is the heat map, displays the trajectory of zebrafish movement within the same timeframe. Supplementary Table 1:Clinical characteristics and sample details of the nine enrolled patients. Supplementary Table 2: List of primers used in RT-qPCR.

## Data Availability

The data that support the findings of this study are available from the corresponding author upon reasonable request.
