# Supplementary material for: Somatic PDGFRB activating variants promote smooth muscle cell phenotype modulation in intracranial fusiform aneurysm
Source: J Biomed Sci. 2024 May 13;31:51. doi: 10.1186/s12929-024-01040-7 (PMC11092182; doi:10.1186/s12929-024-01040-7)
Supplement: Supplementary file 7 — Supplementary Material 7: Supplementary Figure 1: Imaging information of 9 patients with intracranial aneurysms (All fusiform aneurysms except AN-05). Supplementary Figure 2:The workflow of Whole-exome sequencing. Supplementary Figure 3: Details of the four mutations located within the PDGFRB gene region. Supplementary Figure 4:Starvation treatment and PDGFBB stimulation. Supplementary Figure 5:Sanger sequencing results of AN-08 and AN-09. Supplementary Figure 6:The infiltration of microglia around the head vasculature in zebrafish. The left side shows the statistical results of microglial cell counts around the blood vessels in zebrafish from each treatment group. The right side shows the infiltration of microglial cells around the head vasculature in zebrafish from each treatment group (red represents microglial cells, green represents blood vessels). Supplementary Figure 7:5-day post-fertilization (dpf) zebrafish locomotor activity: Left panel shows the distance traveled by zebrafish within one hour; Right panel is the heat map, displays the trajectory of zebrafish movement within the same timeframe. Supplementary Table 1:Clinical characteristics and sample details of the nine enrolled patients. Supplementary Table 2: List of primers used in RT-qPCR. [file 12929_2024_1040_MOESM7_ESM.pdf]

Supplementary Figure 1: Imaging information of 9 patients with intracranial aneurysms (All fusiform aneurysms except AN-05)

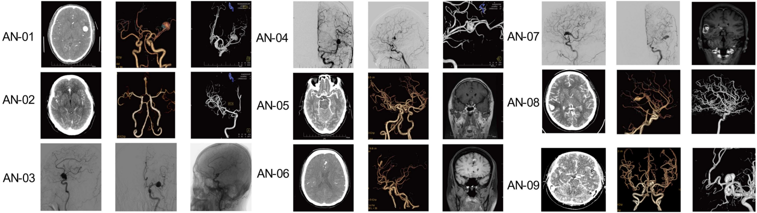

Supplementary Figure 2: The workflow of Whole-exome sequencing

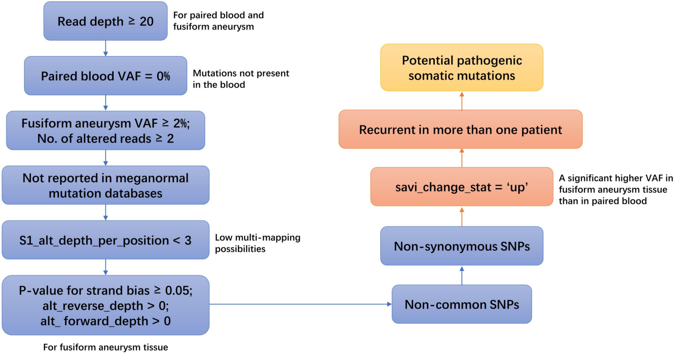

Supplementary Figure 3: Details of the four mutations located within the PDGFRB gene region

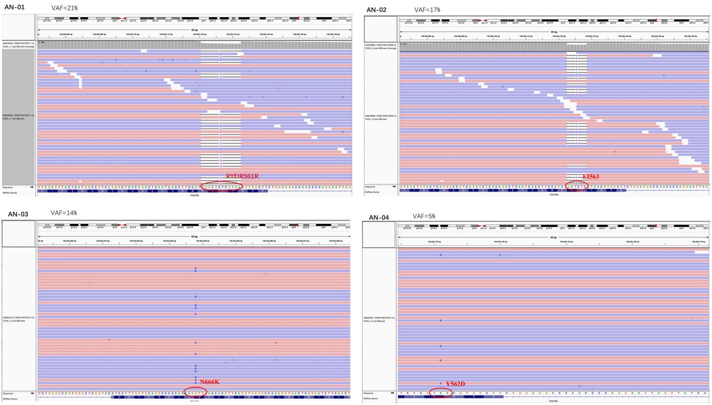

Supplementary Figure 4: Starvation treatment and PDGFBB stimulation

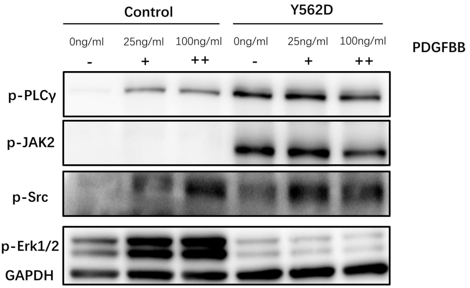

Supplementary Figure 5: Sanger sequencing results of AN-08 and AN-09

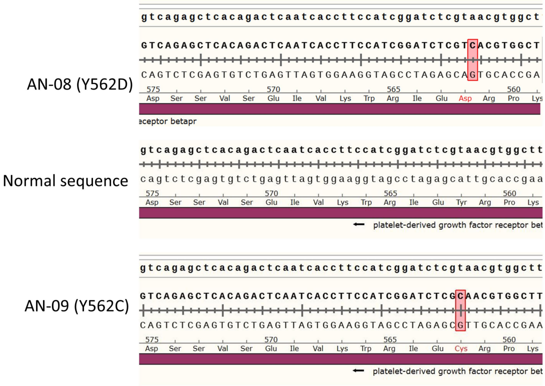

Supplementary Figure 6: Effects of overexpression of PDGFRB on the number of microglia in zebrafish

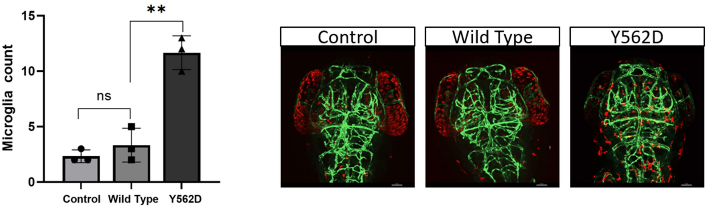

Supplementary Figure 7: Effects of overexpression of PDGFRB on movement patterns of zebrafish

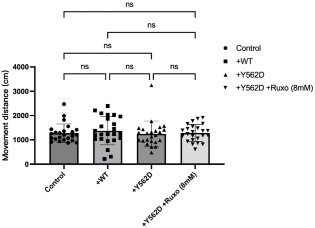

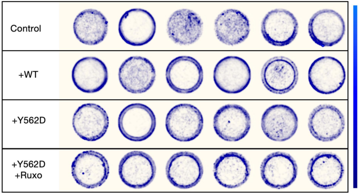

Supplementary Table 1: Clinical characteristics and sample details of the eight enrolled patients

Case ID	Age	Sex	Application	Shape	Location	Symptom	Hypertension	Diabetes	Hyperlipemia	Smoking	Drinking
AN-01	41	Male	WES/IF	Fusiform	MCA	Trauma	No	No	No	No	No
AN-02	44	Female	WES/IF	Fusiform	ACA	SAH	Yes	No	No	No	No
AN-03	66	Male	WES/IF	Fusiform	MCA	Infarction	Yes	No	No	Yes	No
AN-04	29	Female	WES/IF	Fusiform	MCA	Headache	No	No	No	No	No
AN-05	68	Male	WES	Saccular (Thrombus)	AcoA	Dizziness	No	No	No	No	No
AN-06	51	Female	WES/IF	Fusiform (Thrombus)	ACA	Headache	No	No	No	No	No
AN-07	59	Female	WES	Fusiform (Dissection)	MCA	ICH	Yes	No	Yes	No	No
AN-08	24	Female	Single-cell/LCM	Fusiform	MCA	Headache	No	No	No	No	No
AN-09	26	Male	Single-cell/Sanger	Fusiform	ACA	Headache	No	No	No	No	Yes

WES: Whole-exome sequencing; IF: Immunofluorescence; LCM: Laser capture microdissection; Single-cell: Single-cell sequencing; MCA: Middle cerebral artery; ACA: Anterior cerebral artery; AcoA: Anterior communicating artery; SAH: Subarachnoid hemorrhage; ICH: intracranial hematoma

Supplementary Table 2: List of primers used in RT-qPCR

**ACTA2**	Forward	GTGTTGCCCCTGAAGAGCAT
	Reverse	GCTGGGACATTGAAAGTCTCA
**TAGLN**	Forward	AGTGCAGTCCAAAATCGAGAAG
	Reverse	CTTGCTCAGAATCACGCCAT
**ICAM1**	Forward	TTGGGCATAGAGACCCCGTT
	Reverse	GCACATTGCTCAGTTCATACACC
**VCAM1**	Forward	GGGAAGATGGTCGTGATCCTT
	Reverse	TCTGGGGTGGTCTCGATTTTA
**MMP1**	Forward	GGGGCTTTGATGTACCCTAGC
	Reverse	TGTCACACGCTTTTGGGGTTT
**MMP9**	Forward	TGTACCGCTATGGTTACACTCG
	Reverse	GGCAGGGACAGTTGCTTCT
